# Osteoarthritis after major combat trauma: the Armed Services Trauma Rehabilitation Outcome Study

**DOI:** 10.1093/rap/rkaf033

**Published:** 2025-03-14

**Authors:** Fearghal P Behan, Alexander N Bennett, Fraje Watson, Susie Schofield, Eleanor F Miller, Oliver O’Sullivan, Christopher J Boos, Nicola T Fear, Paul Cullinan, Philip G Conaghan, Anthony M J Bull, ADVANCE Study, Maria-Benedicta Edwards, Helen Blackman, Melanie Chesnokov, Emma Coady, Sarah Evans, Guy Fraser, Meliha Kaya-Barge, Maija Maskuniitty, David Pernet, Helen Prentice, Urszula Pucilowska, Stefan Sprinckmoller, Lajli Varsani, Anna Verey, Molly Waldron, Danny Weston, Tass White, Seamus Wilson, Louise Young, Dan Dyball, Ian Gibb, David Gray, Edward Sellon

**Affiliations:** Department of Bioengineering, Imperial College London, London, UK; Discipline of Physiotherapy, Trinity College Dublin, Dublin, Ireland; Academic Department of Military Rehabilitation, Defence Medical Rehabilitation Centre, Stanford Hall, Loughborough, UK; National Heart and Lung Institute, Imperial College London, London, UK; Department of Bioengineering, Imperial College London, London, UK; National Heart and Lung Institute, Imperial College London, London, UK; Department of Bioengineering, Imperial College London, London, UK; Academic Department of Military Rehabilitation, Defence Medical Rehabilitation Centre, Stanford Hall, Loughborough, UK; Academic Department of Military Rehabilitation, Defence Medical Rehabilitation Centre, Stanford Hall, Loughborough, UK; Academic Unit of Injury, Recovery and Inflammation Science, School of Medicine, University of Nottingham, Nottingham, UK; Department of Cardiology, University Hospital Dorset, NHS Trust, Poole, UK; Academic Department for Military Mental Health, King’s College London, London, UK; National Heart and Lung Institute, Imperial College London, London, UK; Leeds Institute of Rheumatic and Musculoskeletal Medicine, University of Leeds, and National Institute for Health and Care Research Leeds Biomedical Research Centre, Leeds, UK; Department of Bioengineering, Imperial College London, London, UK

**Keywords:** Knee osteoarthritis, knee injury, amputation, major trauma

## Abstract

**Objective:**

To investigate the differences in clinical and radiographic knee OA markers between injured and uninjured UK service personnel.

**Methods:**

This study was a cross-sectional analysis, 8 years post-injury, of a prospective cohort study. The Knee Injury and Osteoarthritis Outcome Scores (KOOS), radiographic Kellgren and Lawrence (KL) scores and Osteoarthritis Research Society International scores (joint space narrowing, sclerosis, osteophytes) were obtained from 565 uninjured and 579 matched (on sex, age, rank, regiment and role on deployment) major combat injured participants from the Armed Services Trauma Rehabilitation Outcome study; 35 had a knee injury and 142 had an amputation without knee injury. Kruskal–Wallis tests were used to compare between groups for KOOS and radiographic measures. A multiple logistic regression was performed on the effects of injury on radiographic features.

**Results:**

The mean age at injury was 25.7 years (s.d. 5.2). Injured participants demonstrated worse KOOS values for pain {median 89 [interquartile range (IQR) 72–100] *vs* 94 [83–100]} and symptoms [median 80 (IQR 60–90) *vs* 85 (70–95), *P* < 0.001] and higher scores for radiographic variables than uninjured participants. Injured non-amputated/non-knee-injured participants had worse KOOS values than uninjured participants [pain: 92 (IQR 75–100) *vs* 94 (83–100); symptoms: 80 (IQR 60–90) *vs* 85 (70–95), *P* < 0.01]. Knee-injured participants had worse KOOS values [pain: 67 (IQR 55–85), symptoms: 55 (IQR 35–73), *P* < 0.001] than all subgroups and worse radiographic measures than injured non-amputated participants. KL score (≥1) and sclerosis were worse for amputees than injured non-amputated participants. Amputees had 4.04-fold increased odds (95% CI 2.45, 6.65) *vs* uninjured participants and knee-injured participants had 4.06-fold increased odds (95% CI 1.89–8.74) than uninjured participants of knee osteoarthritis (KOA; KL ≥1). Injured participants (without knee injury/amputation) had 1.74-fold (95% CI 1.27, 2.69) increased odds of KOA than uninjured participants.

**Conclusion:**

Major combat trauma (in addition to knee injury or amputation) has a substantial effect on the development of KOA.

Key messages

- Individuals with a knee injury or amputation had >4-fold higher odds of radiographic KOA than uninjured individuals.
- Major battlefield trauma, without a knee injury or amputation, also increases the odds of KOA.
- Younger individuals post-amputation may be an important population to target for KOA preventative measures to reduce disability.

## Introduction

OA results in significant pain, disability and a reduction in quality of life [1]. The initiation and progression of OA is associated with elevated loading [2], genetic factors [3] and trauma [4], including meniscal, ligamentous and capsular tears, as well as joint dislocation and intra-articular fracture [5]. Sufferers of post-traumatic knee OA (KOA) present with symptoms, on average, 10 years earlier than those with idiopathic KOA [5]. This increased OA risk associated with trauma may be mediated by reduced stability, inflammation and the sequelae of biologic mediators (cytokines, proteolytic enzymes, reactive oxygen species etc) [1], as well as changes to the joint mechanics that the trauma has induced [6, 7].

Much of the literature in post-traumatic OA has focused on sporting injuries, often decades after the original injury [4], with less understanding of the short-term effects of trauma. People with traumatic amputations have been reported to have an increased risk of medial KOA in the intact limb >25 years from amputation [8, 9]. Increased medial knee joint contact forces on the intact limb have been demonstrated [10], suggesting that this increased KOA is related to biomechanical factors. However, how quickly this altered loading can result in joint symptoms or structural changes in amputated individuals is currently unknown.

There is also a paucity of data on the effects of major trauma, such as combat blast trauma, on the development of KOA. The Armed Services Trauma Rehabilitation Outcome (ADVANCE) study is investigating the long-term outcomes of combat casualties from the Afghanistan War (>500 injured participants and >500 matched non-injured participants) and is the first prospective cohort study of its kind [11]. The injured cohort consists of a variety of combat injuries, including blast injuries, gunshot wounds, traumatic amputation and local knee injuries. This study provides a unique opportunity to investigate the short-, medium- and long-term effects of major trauma on the development of KOA in a large, young cohort with a matched uninjured comparison group.

The current study aims to investigate the association between major combat injuries and the clinical and radiographic outcomes of KOA among UK service personnel in the ADVANCE cohort. We hypothesized that major combat injuries would be associated with an increased risk of both clinical and radiographic KOA.

## Methods

### Study design and participants

The ADVANCE study is a longitudinal cohort study investigating the long-term effects of sustaining a combat injury on physical and psychosocial well-being. This study complies with the Declaration of Helsinki and was approved by the Ministry of Defence Research Ethics Committee (protocol no. 357.PPE/12). A total of 579 physically injured UK male military personnel and 565 uninjured personnel frequency matched to the injured group on sex, age, rank, regiment, role on deployment, service and deployment era were recruited from a sample provided by the Ministry of Defence, Defence Statistics (UK) [11]. Eligibility criteria for the ‘injured’ group included having sustained a physical combat injury during deployment to Afghanistan and having an aeromedical evacuation due to the injury that resulted in admission to a UK hospital. Both needed to be satisfied for inclusion. These injuries could all be defined as ‘major trauma’ [12]. Eligibility criteria for the ‘uninjured’ group included having deployed to Afghanistan but sustaining no physical combat injuries. Full inclusion and exclusion criteria can be found in the ADVANCE protocol paper [11]. Females were excluded due to an insufficient number of injured females for statistical power. Participants in this cohort underwent an initial cross-sectional assessment as part of the ADVANCE study 8 years on average after their initial qualifying injury. The outcomes captured during this assessment were considered to reflect the health state of the individual at that time.

### Procedure

Participants were invited to a study day at the UK Defence Medical Rehabilitation Centre Headley Court (2015–2018) or Stanford Hall (2018–2020). Following written informed consent, participants took part in a comprehensive set of health tests, including clinical assessments, a research nurse-led clinical interview and self-report questionnaires [11].

Diagnoses of the battlefield injury from the trauma records of participants were searched to establish any local knee injury. International Classification of Diseases, Tenth Revision codes involving ‘injury to the knee and lower leg’ (S80–89) were searched manually (in 2023) and if any of the diagnoses were confirmed as a knee injury (e.g. intra-articular fracture, meniscal tear, anterior cruciate knee ligament injury) these participants were subcategorized as ‘knee injured’. If a participant had both an amputation and a knee injury, he/she was categorized as ‘knee injured’ to prevent the effects of the knee injury contaminating the isolated effects of amputation that were hypothesized to be less severe. All injured participants without a knee injury or amputation were considered as ‘injured non-amputated’. Only service personnel with combat-related injuries that required aeromedical evacuation were recruited to the study; non-combat-related injuries were excluded. Consequently, groups were categorized as follows: uninjured (UI) or injured [non-amputated (INA), amputated (IA), knee injury (KI)].

### Self-reported outcomes

The Knee injury and Osteoarthritis Outcome Score (KOOS) is a patient-reported outcome measure widely used in clinical research and practice to assess the consequences of knee disorders in individuals with a knee injury and/or KOA [13]. KOOS has high test–retest reliability [14], proven convergent and divergent construct validity [13] and is responsive to change [15]. KOOS has subscales scored separately from 0 (extreme knee problems) to 100 (no knee problems). The KOOS pain and symptoms subscales are reported in this analysis and presented as median [interquartile range (IQR)].

### Radiographic assessment

**Figure 1.**
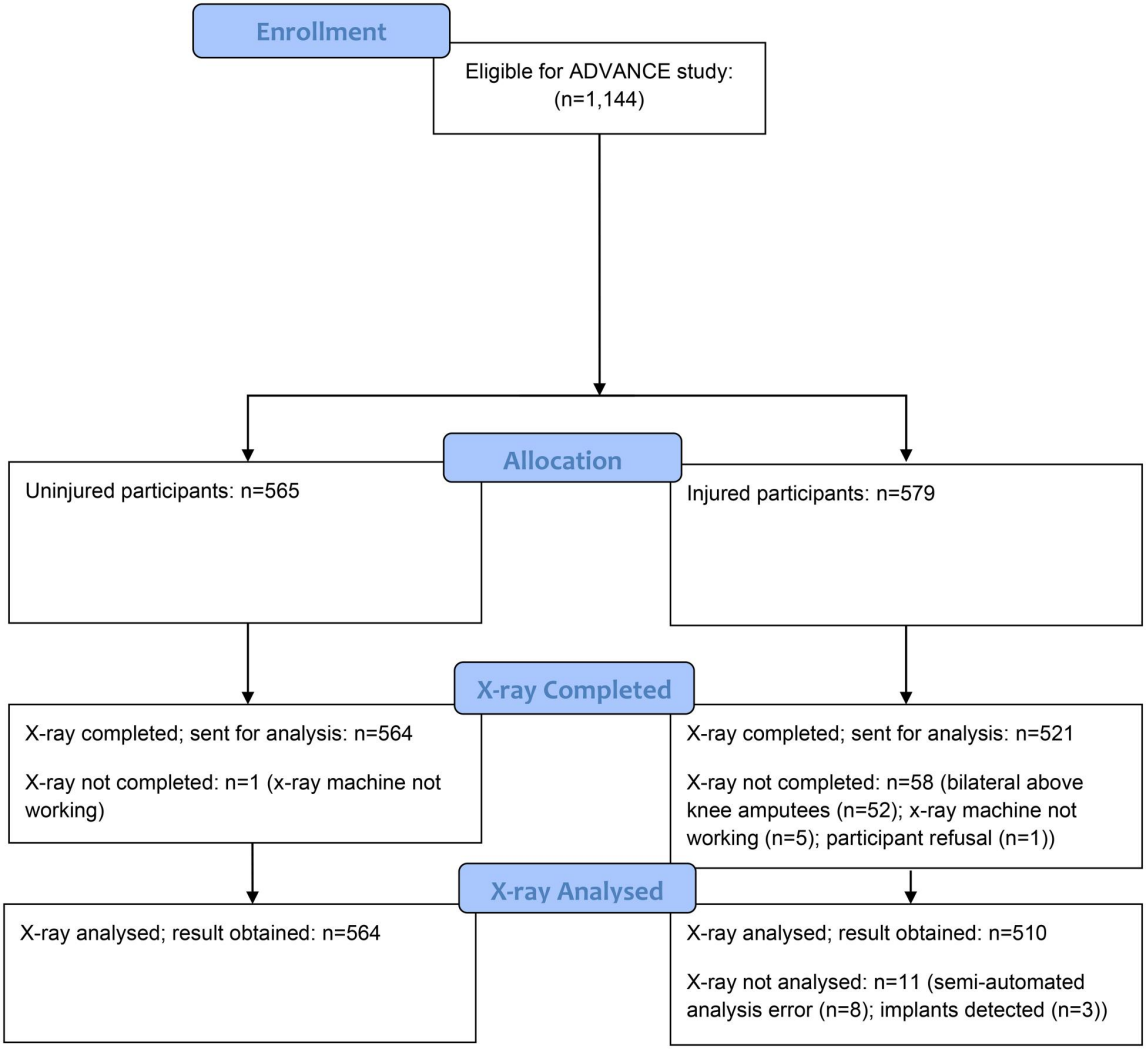
Flow diagram for radiographic assessment.

Posterior–anterior views with the knees in a semiflexed position (7–10°) using the Synaflexer radiograph positioning frame (Synarc, San Francisco, CA, USA) [16] were performed as per recommendations for the assessment of KOA [11, 17]. Radiographs were performed on all possible participants’ knees. The established reasons for missing radiographs are presented in Fig. 1. The radiographs were analysed with the Knee Osteoarthritis Labelling Assistant (KOALA; IB Lab, Vienna, Austria) automated measurement software with manual checking. KOALA was developed using deep learning algorithms and trained on a large dataset of individual radiographs of the knee; this method has shown excellent accuracy values for receiver operating characteristics curves [18, 19]. The following radiographic scoring measures of KOA within the tibiofemoral joint were obtained: Kellgren–Lawrence (KL), Osteoarthritis Research Society International (OARSI) joint space narrowing (JSN), OARSI sclerosis and OARSI osteophyte scales. The KL classification system grades OA severity from grade 0, representing an absence of OA changes on radiographs, to grade 4, representing severe OA changes on radiographs [20]. The three OARSI scales are graded from grade 0, representing an absence of OA changes on radiographs, to grade 3, representing severe OA changes on radiographs [21].

### Statistical analysis

Statistical analysis was carried out using Stata version 16 (StataCorp, College Station, TX, USA). Data were screened for normality using the Shapiro–Wilk test. As most participants (non-amputated and below-knee amputations) had two values for each variable (right and left knee), the value demonstrating more advanced signs of KOA was utilized for analysis (i.e. lowest KOOS value, highest radiographic value). For participants with above-knee amputations and only a single knee joint intact for analysis, we utilized this singular value. For comparisons between two groups (uninjured *vs* injured), Mann–Whitney U tests were used. For comparisons between three or more groups (UI *vs* INA *vs* IA *vs* KI) the Kruskal–Wallis test was used, with Dunn’s post hoc used for group comparisons where a difference was found. All comparisons between subcategories were controlled for multiple comparisons to reduce the chance of type 1 error. Logistic regression was used to assess the relationship between injury status overall (injured, uninjured) and imaging status. Imaging status was quantified as a KL of 0 (no signs of KOA on radiographs) or any KL score ≥1. A second regression was undertaken with imaging status defined as KL <2 or KL ≥2, using a stricter definition of KOA. Based on the literature, a priori confounding variables of age and socio-economic status, defined using the National Statistics Socio-economic Classification as military rank [22–24] and split into junior rank (OR 1–4), senior rank (OR 5–9) and officer rank (OF 1–9), were controlled for in the model. Although many of our measured variables (ethnicity, BMI, smoking etc) are associated with the outcome of interest (OA), they are not independently associated with the exposure (injury) and thus are not considered true confounders. Analysis was repeated for the subcategories of injury (UI, INA, IA and KI). Multiple imputation was considered for the missing data. However, since data were only missing (*n *=* *70; Fig. 1) on the outcome, then complete case and multiple imputation would provide equivalent results [25]. Therefore, we present a complete case analysis only.

## Results

### Participant demographics

Knee radiographs were obtained in 564 (99.8%) uninjured participants and 510 (88.0%) injured participants (395 INA, 84 IA, 31 KI). Participants had a mean age at assessment of 34 years (s.d. 5) and the injured participants were a mean of 8 years (range 1–14) post-injury. Full demographics at the baseline assessment can be found in Table 1. No significant differences were found between groups for age or height (*P* > 0.05). Injured amputated participants had a higher adjusted BMI than uninjured participants (*P* < 0.01; Table 1) and knee-injured participants had a higher adjusted BMI than uninjured and injured non-amputated participants (*P* < 0.01). There was no difference between knee injuries prior to exposure between groups (9.2% of the uninjured group, 7.1% of the injured group; χ^2^ = 1.72, *P* = 0.189). The injured group had a larger percentage of those of junior rank, while the uninjured had a larger percentage of those of senior or officer rank. No differences were found for clinical and radiographic measures between injury mechanisms (blast *vs* gunshot/non-blast; data not presented) and are therefore presented together in the injured group.

**Table 1.** Demographics for uninjured and injured (non-amputated and amputated) at baseline testing

Characteristics	Uninjured (*n *=* *565)	All injured (*n *=* *579)	Injured: non-amputated (*n *=* *402)	Injured: amputated (*n *=* *142^b^)	Injured: knee injury (*n *=* *35)
Age at deployment/injury, years, mean (s.d.)	26.5 (5.3)	25.7 (5.2)	25.8 (5.3)	25.5 (4.7)	26.1 (5.1)
Age at assessment, years, mean (s.d.)	34.2 (5.4)	34.0 (5.3)	34.3 (5.5)	33.1 (4.7)	33.9 (5.6)
Time from injury to assessment, years, mean (s.d.)	–	8.3 (2.1)	8.6 (2.2)	7.6 (1.9)	7.8 (2.3)
Height (cm), mean (s.d.)	178.9 (6.4)	179.3 (7.1)	179.0 (6.7)	180.4 (8.1)	178.3 (7.0)
BMI (adjusted for injured^a^, kg/m^2^), mean (s.d.)	27.4 (3.4)	28.1 (3.9)	27.8 (3.5)	28.7 (4.4)	30.0 (4.9)
Sampling rank/SEC, *n* (%)					
Officer rank	79 (14.0)	59 (10.2)	44 (10.9)	13 (9.1)	2 (5.7)
Senior rank	147 (26.0)	106 (18.3)	82 (20.3)	18 (12.7)	6 (17.1)
Junior rank	339 (60.0)	414 (71.5)	276 (68.7)	111 (78.2)	27 (77.1)
Caucasian, *n* (%)	512 (90.6)	524 (90.5)	361 (89.8)	129 (90.8)	34 (97.1)
Still serving in military, *n* (%)	466 (82.5)	158 (27.3)	139 (34.6)	15 (10.6)	4 (11.4)

a Adjusted for amputees to account for limb loss [26].

b 15 amputees included in knee-injury group, 157 amputees overall.

SEC: socio-economic classification.

### KOOS

#### Uninjured vs injured

**Figure 2.**
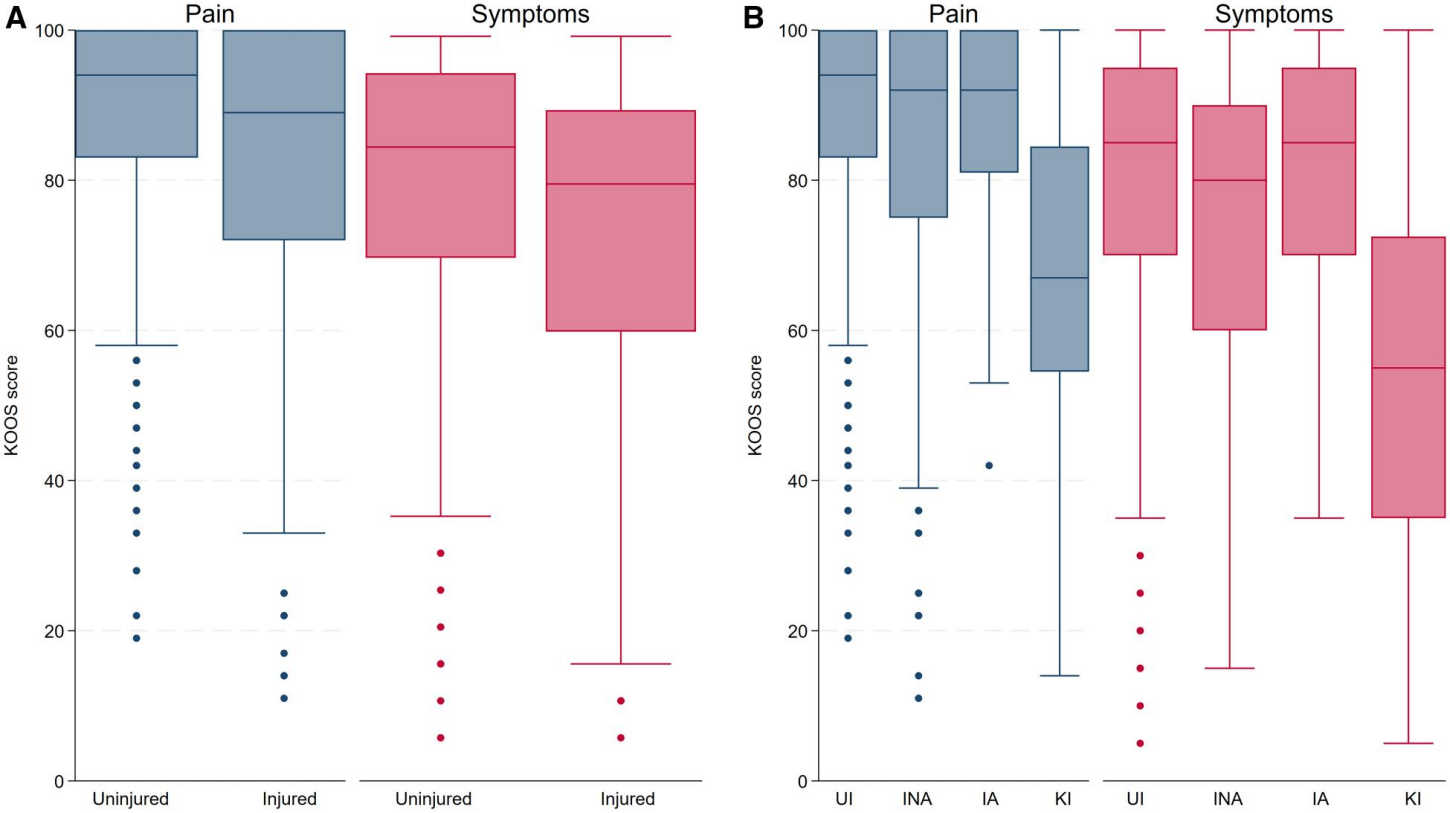
KOOS pain and symptoms for **(A)** uninjured *vs* injured, **(B)** UI *vs* INA *vs* (IA) *vs* KI. (A): *n* = 563 and 521, (B): *n* = 563, 396, 93 and 32, respectively.

Injured participants demonstrated lower (worse) median values for both the pain and symptoms subscales of the KOOS than the uninjured group [median 89 (IQR 72–100) *vs* 94 (83–100) and 80 (IQR 60–90) *vs* 85 (70–95), respectively; *P* < 0.001; Fig. 2A], indicating worse knee pain and symptoms.

#### UI vs INA vs IA vs KI

There was a difference between groups for both the pain and symptoms subscales (*P* < 001), with INA participants demonstrating lower values than UI participants on both subscales [pain: 92 (IQR 75–100) *vs* 94 (83–100); symptoms: 80 (IQR 60–90) *vs* 85 (70–95); *P* < 0.01; Fig. 2B], indicating worse knee pain and symptoms. The IA participants did not differ from UI participants on either subscale. The INA participants demonstrated lower values for symptoms than IA participants [80 (IQR 60–90) *vs* 85 (70–95); *P* < 0.05], indicating worse knee symptoms. The KI group demonstrated lower values [pain: 67 (IQR 55–85); symptoms: 55 (IQR 35–73)] than all other groups for both subscales (*P* < 0.001; Fig. 2B), indicating that this group had worse knee pain and symptoms than all other groups.

### Radiographic measures

#### Uninjured vs injured

**Figure 3.**
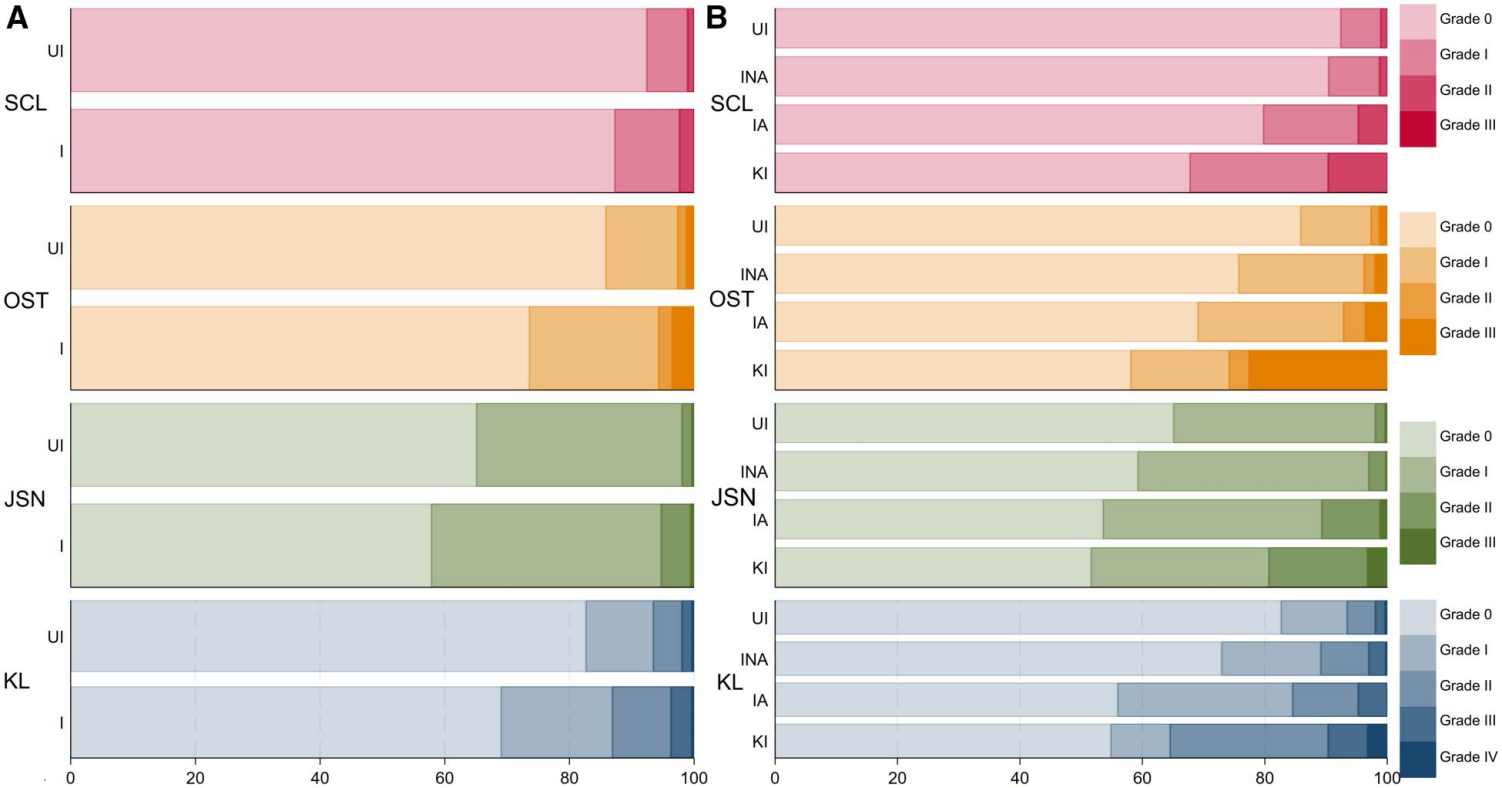
Radiographic results for **(A)** uninjured and injured and **(B)** UI, INA, IA and KI. Participants percentage of each grade for each imaging subscale. (A): *n* = 564 and 510, (B) *n* = 564, 395, 84, and 31, respectively. OST: osteophytes; SCL: sclerosis.

Injured participants demonstrated significantly higher imaging grades, suggesting more advanced signs of KOA, than uninjured participants in all imaging measures (*P* < 0.01; Fig. 3A).

#### UI vs INA vs IA vs KI

There was a significant difference on all radiographic scales between groups (*P* < 0.005; Fig. 3B). Post hoc analyses demonstrated higher imaging grades in INA participants than UI participants for KL and osteophytes (*P* < 0.05; Fig. 3B). The IA participants demonstrated higher imaging grades than UI participants for all measures (*P* < 0.05; Fig. 3B) and higher imaging grades than INA participants for KL and sclerosis (*P* < 0.01; Fig. 3B). KI participants demonstrated worse imaging grades than both UI and INA participants for KL, osteophytes and sclerosis (*P* < 0.05; Fig. 3B), but no differences compared with IA participants.

#### Regression analysis

Participants who were injured had increased odds of KL ≥1 by a factor of 2.13 (95% CI 1.59, 2.87) compared with uninjured participants, after controlling for confounders. Participants who experienced an amputation had increased odds of KL ≥1 by a factor of 4.04 (95% CI 2.45, 6.65) and participants who sustained a knee injury by a factor of 4.06 (95% CI 1.89, 8.74) compared with those who were uninjured (Table 2). Injured participants who sustained neither a knee injury nor an amputation had increased odds of KL ≥1 on knee radiographs by a factor of 1.74 (95% CI 1.27, 2.40) compared with those who were uninjured (Table 2).

**Table 2.** Odds ratios (ORs) from logistic regressions of two knee radiographic imaging outcomes (KL ≥1 and KL ≥2) for injury status overall (uninjured, injured) and injury status subcategories (UI, INA, IA and KI).

Predictor variable	Injury status overall			Injury status subcategories	
	Unadjusted OR (95% CI)	Adjusted OR (95% CI)	*P*-value	Adjusted OR (95% CI)	*P*-value
Outcome 1: KL ≥1					
Injury status (overall)					
Uninjured	1 (ref)	1 (ref)	<0.001		
Injured	2.13 (1.60, 2.84)	2.13 (1.59, 2.87)			
Injury status (subcategories)					
UI	1 (ref)			1 (ref)	<0.001
INA	1.77 (1.29, 2.41)			1.74 (1.27, 2.40)	
IA	3.74 (2.31, 6.07)			4.04 (2.45, 6.65)	
KI	3.92 (1.87, 8.21)			4.06 (1.89, 8.74)	
Confounders					
Age	1.09 (1.06, 1.12)	1.10 (1.07, 1.14)	<0.001	1.11 (1.07, 1.14)	<0.001
Socio-economic status					
Junior rank	1 (ref)	1 (ref)	0.072	1 (ref)	0.108
Mid rank	1.15 (0.81, 1.61)	0.69 (0.47, 1.03)		0.72 (0.48, 1.07)	
Officer rank	1.08 (0.70, 1.66)	0.71 (0.44, 1.16)		0.75 (0.46, 1.22)	
Outcome 2: KL ≥2					
Injury status (overall)					
Uninjured	1 (ref)	1 (ref)	<0.001		
Injured	2.15 (1.41, 3.28)	2.11 (1.37, 3.24)			
Injury status (subcategories)					
UI	1 (ref)			1 (ref)	<0.001
INA	1.74 (1.10, 2.76)			1.68 (1.05, 2.68)	
IA	2.61 (1.32, 5.14)			2.72 (1.35, 5.45)	
KI	7.83 (3.49, 17.57)			8.15 (3.53, 18.82)	
Confounders					
Age	1.08 (1.05, 1.13)	1.10 (1.07, 1.15)	<0.001	1.11 (1.06, 1.16)	<0.001
Socio-economic status					
Junior rank	1 (ref)	1 (ref)	0.128	1 (ref)	0.158
Senior rank	1.11 (0.68, 1.80)	0.65 (0.37, 1.13)		0.66 (0.38, 1.17)	
Officer rank	1.06 (0.57, 1.98)	0.66 (0.33, 1.32)		0.70 (0.35, 1.41)	

Using the stricter radiographic definition of KOA, participants who were injured had increased odds of KL ≥2 on knee radiographs [odds ratio 2.11 (95% CI 1.37, 3.24)] compared with uninjured participants, after controlling for confounders. When comparing subcategories, participants who experienced an amputation had increased odds of KL ≥2 on knee radiographs by a factor of 2.72 (95% CI 1.35, 5.45), as did participants who sustained neither a knee injury nor an amputation, by a factor of 1.68 (95% CI 1.05, 2.69), compared with those who were uninjured (Table 2). Injured participants who sustained a knee injury had increased odds of KL ≥2 on knee radiographs by a factor of 8.15 (95% CI 3.53, 18.82) compared with those who were uninjured, after controlling for confounders (Table 2).

## Discussion

This baseline analysis of the ADVANCE cohort study demonstrated that major combat trauma is associated with an increase in both clinical and radiographic signs of KOA compared with matched uninjured participants. Individuals who experienced a knee injury or an amputation experienced more than a 4-fold increase in the odds (KI: 95% CI 1.89, 8,74; IA: 95% CI 2.45, 6.65) of KOA radiographic changes (KL ≥1) compared with uninjured participants within ≈8 years post-injury. These findings concur with previous literature demonstrating increased KOA after knee injury sustained during military deployment [27, 28], however, this study presents data on a younger population than previous retrospective studies showing OA risk post-trauma [29], a shorter time from injury to assessment than previous findings [4], in a large cohort, compared with a matched comparison group, and demonstrates an increased KOA risk without local knee injury [27, 28]. Those who experienced major combat trauma without an amputation or local knee injury had a 1.74-fold (KL ≥1) or 1.68-fold (KL ≥2) increase in the odds of knee degeneration on radiographs compared with uninjured individuals. This is the first study to show the association of increased KOA with generalized major trauma without a local knee injury or amputation.

Our patient-reported outcome measure, the KOOS, demonstrated poorer results in our injured group compared with our uninjured group, as did our radiographic findings. However, subcategorizing our injured participants demonstrates interesting findings for the KOOS compared with our radiographic results. Although the knee-injured group demonstrated worse radiographic findings alongside worse KOOS scores, this relationship was more nuanced for the amputated injured and non-amputated injured groups. The amputated participants demonstrated no difference in their self-reported pain and symptoms compared with the uninjured population despite worse radiographic findings on all scales (KL, JSN, osteophytes, sclerosis). Injured non-amputated participants reported worse pain and symptoms than the uninjured participants alongside a higher rate of KOA changes on imaging. As the time from injury to our findings is relatively short, 8 years on average, the pattern of these outcomes within the ADVANCE follow-up assessments in these subcategories will provide further insights on the relationship between imaging findings and self-reported pain and symptoms.

The negative effects of knee trauma on KOA are clearly apparent within 8 years in this cohort. The participants who had sustained a knee injury had the worst self-reported pain and symptoms and the highest proportion of KOA on radiographs compared with other groups (UI, INA and IA). This reinforces previous literature on increased KOA risk after general knee trauma [4, 29], systematic reviews demonstrating increased KOA following a variety of knee injuries [30, 31] and increased knee OA risk after knee injuries sustained during military deployment [27, 28]. The local pathology after knee trauma is believed to be initiated by intra-articular pathogenic processes, such as subchondral bone remodelling, cellular infiltration, the release of inflammatory mediators in synovial fluid and apoptosis of articular chondrocytes [32], alongside local joint instability and altered joint mechanics [1]. Our findings of increased radiographic signs of KOA within our amputee participants also supports previous literature [8, 9]. The amputated participants had a higher BMI than the uninjured participants—higher BMI is associated with higher OA pain [33]—which may have augmented this outcome.

Although our findings are consistent with similar research regarding the effects of knee trauma and amputation on knee OA, the effect of battlefield injury on knee OA risk without an associated amputation or local knee injury is less clear from previous literature. The most novel finding from this study demonstrated that injured participants who had neither an amputation nor local knee injury had increased OA radiographic changes, for both KL and OARSI osteophyte subscales, compared with our uninjured participants. Their odds of OA on knee radiographs were increased compared with our uninjured participants, indicating a possible systemic effect of major trauma on KOA. This group also reported worse pain and symptoms than the uninjured participants.

Our injured cohort were on average 34 years old and ≈8 years post-injury. This is a younger population with a shorter follow-up time than previous studies reporting increased signs of OA after knee injury [4, 34, 35]. Previous military studies had similar ages and follow-up times [27, 28], however, these studies had no matched comparison group, no patient-reported outcome measures, nor were amputees analysed separately [27, 28]. This allowed us to investigate the effects of amputation on knee degeneration on radiographs in isolation by removing the effects of knee injury. Our amputated participants had increased odds of KOA compared with our uninjured participants. This is a novel finding in an amputee population of this age [8, 9] and identifies younger amputees as a potential population to target with preventative strategies to minimize the risk of disability caused by KOA. It must also be acknowledged that our results are compared with matched uninjured military personnel. Military personnel have been found to have a higher risk of KOA compared with the general population [36], therefore future analyses may include comparisons to an age- and sex-matched control group from the general population.

Interpreting these differences clinically may be challenging, but metrics such as the patient acceptable symptoms state (PASS) may be helpful when interpreting a single measure of a patient-reported outcome measure. PASS levels for the KOOS have been reported as 80.5–84.0 for pain and 83.0–87.5 for symptoms in patients after total knee arthroplasty (TKA) [37] and 73.6–76.4 for pain and 71.2–73.2 for symptoms in patients after platelet-rich plasma (PRP) injection treatment for KOA [38]. Any value below these thresholds indicates symptoms are unacceptable to patients. The median pain within our injured cohort (89) remains above these PASS levels, while the injured cohort’s median KOOS symptom levels (80) are above the threshold for KOA patients after PRP injection [38], but below those for post-TKA [37]. Those with a knee injury were below the PASS level for both pain (67) and symptoms (55), while those with no amputation or knee injury were below the post-TKA PASS level for symptoms (80) [37], indicating unacceptable symptoms in both groups. However, amputated participants were above the PASS threshold for both pain and symptoms (92 and 85, respectively).

The inherent structure of this baseline analysis precludes causal inferences; these may be enabled by the prospective follow-up data within the ADVANCE cohort. Furthermore, metabolic influences on joint symptoms and structure have not been examined in the current study but will be the subject of future analyses within the ADVANCE cohort. Although knee injuries were analysed directly, it should be acknowledged that injuries elsewhere in the lower limb may have biomechanical consequences at the knee, potentially altering KOA variables [39]. Time of battle exposure was not controlled for, potentially introducing bias. Although we feel frequency matching was appropriate for the current study, we acknowledge the potential benefits of alternative strategies, such as propensity score matching. Missing data for both clinical and radiographic outcomes may have added further bias. The outcomes regarding the relatively small sample of knee-injured participants, and the resultant large confidence intervals of their results, should be interpreted with care. The lack of statistical power to go through all the subgroups of uni- and bilateral amputations, both above and below the knee, meant that we chose to analyse the knee with the most degeneration for those with both knees intact (bilateral or unilateral transtibial amputees). This limitation means that we were unable to address questions about the interplay between side of injury, amputation side and side of KOA onset.

To conclude, this study is the first to report both radiographic and clinical risk of increased OA after major combat trauma irrespective of knee injury or amputation. Injured participants demonstrated higher rates of knee degeneration and poorer self-reported pain and symptoms compared with uninjured participants. Furthermore, radiographic OA is worse in those with knee injuries, those following amputations and those who were injured without an amputation or knee injury than uninjured participants. Those with knee injuries and injured participants without amputation or knee injury also have worse self-reported pain and symptoms than uninjured participants, while, interestingly, amputated participants report similar pain and symptom levels to uninjured participants. Follow-up assessments are under way as part of the ADVANCE study, and these will facilitate further understanding of these patterns and guide potential future interventional or preventative studies.

## Data Availability

Sharing of anonymous data will be considered by the project board upon reasonable request.
